# Supplementary figures and images for: Circulating exosome-circRNAs mediated downregulation of FGF9 through ceRNA mechanism aggravates renal fibrosis in diabetic nephropathy
Source: PLoS One. 2025 Jun 17;20(6):e0326217. doi: 10.1371/journal.pone.0326217 (PMC12173226; doi:10.1371/journal.pone.0326217)

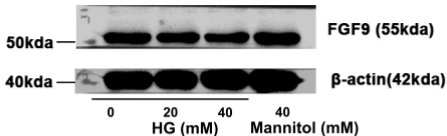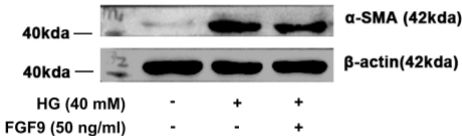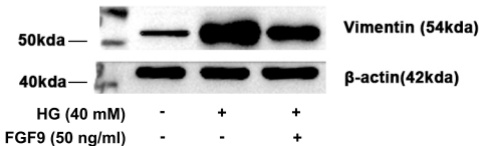

Supplement: S1 Raw Images — (PDF) [file pone.0326217.s001.pdf]

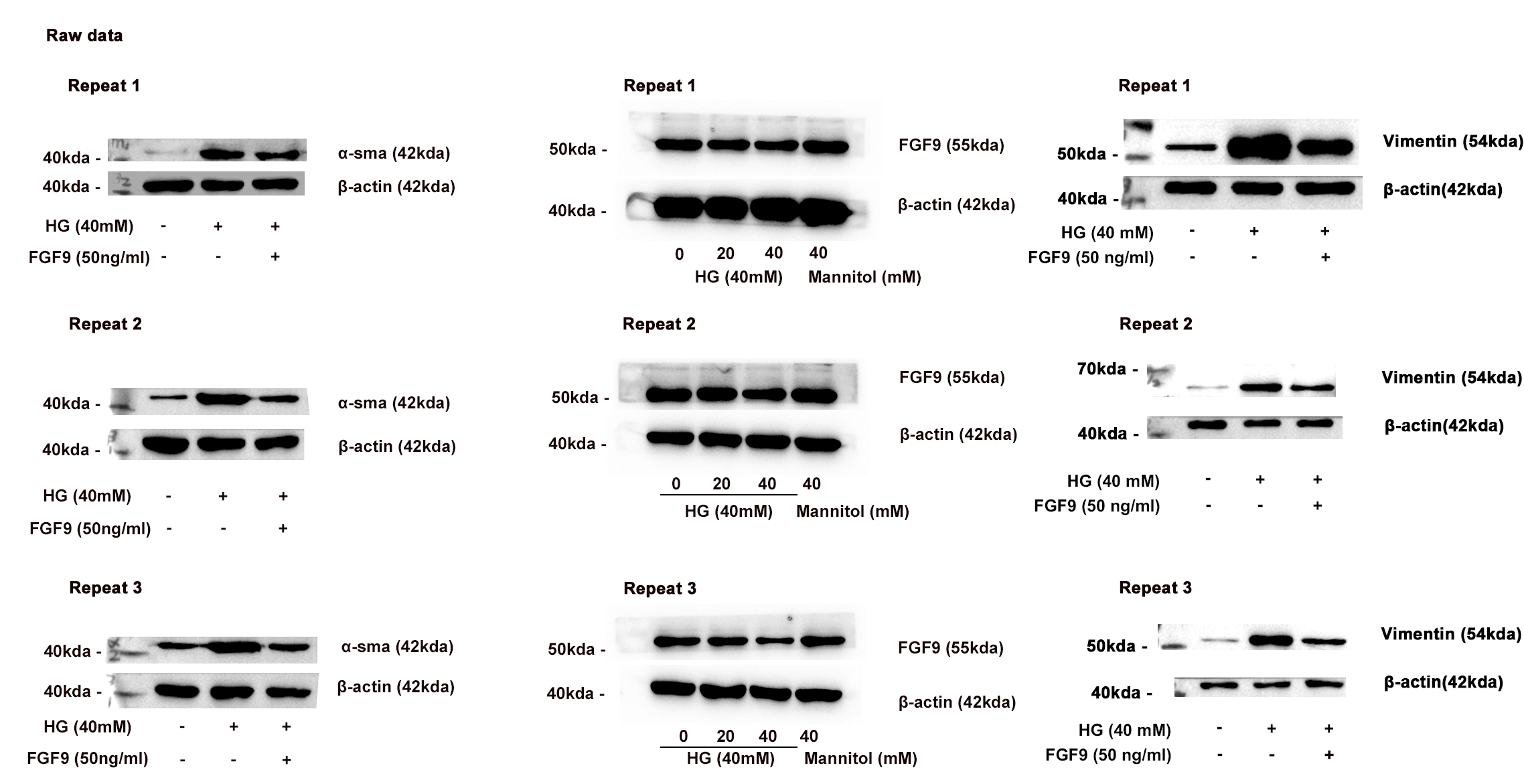

Supplement: S2 File — (TIF) [file pone.0326217.s003.tif]
