# Supplementary material for: Circulating exosome-circRNAs mediated downregulation of FGF9 through ceRNA mechanism aggravates renal fibrosis in diabetic nephropathy
Source: PLoS One. 2025 Jun 17;20(6):e0326217. doi: 10.1371/journal.pone.0326217 (PMC12173226; doi:10.1371/journal.pone.0326217)
Supplement: Attachment — Submitted filename: Rebuttal letter.docx [file pone.0326217.s005.docx]

**Response to reviewers**

We thank the editors and reviewers for the comments on this manuscript (PONE-D-25-08893). The concerns have been addressed as follows:

To Editor

1. **Please ensure that your manuscript meets PLOS ONE's style requirements, including those for file naming. The PLOS ONE style templates can be found at** [**https://journals.plos.org/plosone/s/file?id=wjVg/PLOSOne_formatting_sample_main_body.pdf**](https://journals.plos.org/plosone/s/file?id=wjVg/PLOSOne_formatting_sample_main_body.pdf) **and** **https://journals.plos.org/plosone/s/file?id=ba62/PLOSOne_formatting_sample_title_authors_affiliations.pdf.**

Answer: Thank you very much for reminding. We have thoroughly reviewed our manuscript and ensured that it adheres to PLOS ONE's style requirements as outlined in the provided templates. All file names have been updated to comply with the specified guidelines.

2. **Please state what role the funders took in the study.  If the funders had no role, please state: "The funders had no role in study design, data collection and analysis, decision to publish, or preparation of the manuscript."If this statement is not correct you must amend it as needed.Please include this amended Role of Funder statement in your cover letter; we will change the online submission form on your behalf.**

Answer: Thank you for your guidance regarding the role of the funders in our study. We have carefully reviewed the involvement of the funding sources and have determined that the funders had no role in study design, data collection and analysis, decision to publish, or preparation of the manuscript. We have included the following statement in our cover letter:

"The funders had no role in study design, data collection and analysis, decision to publish, or preparation of the manuscript. "

3. **When completing the data availability statement of the submission form, you indicated that you will make your data available on acceptance. We strongly recommend all authors decide on a data sharing plan before acceptance, as the process can be lengthy and hold up publication timelines. Please note that, though access restrictions are acceptable now, your entire data will need to be made freely accessible if your manuscript is accepted for publication. This policy applies to all data except where public deposition would breach compliance with the protocol approved by your research ethics board. If you are unable to adhere to our open data policy, please kindly revise your statement to explain your reasoning and we will seek the editor's input on an exemption. Please be assured that, once you have provided your new statement, the assessment of your exemption will not hold up the peer review process.**

Answer: Thank you for your important reminder regarding the data availability statement. We appreciate your guidance on this matter. The data supporting our study is already publicly available. The data can be accessed through the following link: https://www.ncbi.nlm.nih.gov/sra/PRJNA1184123, or https://doi.org/10.6084/m9.figshare.28681061.

"**Availability of data and materials:** The sequencing data generated in the present study can be found in the National Center for Biotechnology Information under the login number of the data set: PRJNA1184123 or at the following URL: <https://www.ncbi.nlm.nih.gov/sra/PRJNA1184123>. All relevant data are within the paper and its Supporting Information files. The Supporting Information files have been uploaded to figshare. The direct link to freely access the Supporting Information files is <https://doi.org/10.6084/m9.figshare.28681061>. "

4. **Your ethics statement should only appear in the Methods section of your manuscript. If your ethics statement is written in any section besides the Methods, please delete it from any other section.**

Answer: Thank you for your suggestion. We included the ethic statement only in Methods part..

" All human specimens were obtained from the Affiliated Hospital of Binzhou Medical University from April 22, 2021 to March 11, 2023. The patients were provided written informed consent to participate in this study. The six serum samples were from three healthy people and three DN patients. The criteria for inclusion of healthy people were disease-free. All experiments were all pathologically proven with an estimated glomerular filtration rate (eGFR) no less than 30 ml/min/ 1.73 m2 (based on the Chronic Kidney Disease Epidemiology Collaboration (CKD-EPI) equation). All experiments involving human subjects were approved by the Ethics Committee of Binzhou Medical University (protocol no. 2020-405). "

5. **PLOS ONE now requires that authors provide the original uncropped and unadjusted images underlying all blot or gel results reported in a submission’s figures or Supporting Information files. This policy and the journal’s other requirements for blot/gel reporting and figure preparation are described in detail at https://journals.plos.org/plosone/s/figures#loc-blot-and-gel-reporting-requirements and https://journals.plos.org/plosone/s/figures#loc-preparing-figures-from-image-files. When you submit your revised manuscript, please ensure that your figures adhere fully to these guidelines and provide the original underlying images for all blot or gel data reported in your submission. See the following link for instructions on providing the original image data: https://journals.plos.org/plosone/s/figures#loc-original-images-for-blots-and-gels.**

**In your cover letter, please note whether your blot/gel image data are in Supporting Information or posted at a public data repository, provide the repository URL if relevant, and provide specific details as to which raw blot/gel images, if any, are not available. Email us at plosone@plos.org if you have any questions.**

Answer: Thank you for your suggestion. We have provided all original uncropped and unadjusted images underlying the blot and gel results reported in our figures. These images are included in the Supporting Information files of our revised manuscript.  In our cover letter, we have noted that all blot/gel image data are included in the Supporting Information. We have also provided specific details regarding the availability of each raw blot/gel image, confirming that there are no images that are not available.

6. **Please include captions for your Supporting Information files at the end of your manuscript, and update any in-text citations to match accordingly. Please see our Supporting Information guidelines for more information:** **http://journals.plos.org/plosone/s/supporting-information.**

Answer: Thank you for your suggestion. We have included captions for all Supporting Information files at the end of the manuscript and update any in-text citations to ensure they match the new format as per the Supporting Information guidelines provided by PLOS ONE.

**Reviewer 1:** The manuscript by Yang et al. investigated the mechanistic role of exosome-circRNAs in the exacerbation of renal fibrosis in DN by downregulating the expression of FGF9 through ceRNA mechanism. The authors performed a comprehensive analysis by integrating both dry-lab and wet-lab data to support their claims. However, I found this manuscript had a major defect in their results presentation, which significantly make the results less convincing even though the figures have shown some promising findings. I explain my concerns in more details below.

**1.** **The 'Abstract' section needs to be more concise as the current 'Abstract' described the background and methods in too many details, which will unavoidably make the topic of this study less prominent. I suggest the author shorten the 'Abstract' appropriately.**
Answer: Thank you for your suggestion. We have revised the "Abstrac" according to your suggestion.

" Diabetic nephropathy (DN) is one of the most serious microvascular complications of diabetes mellitus. It is characterized by progressive tubulointerstitial fibrosis. The aim of this study was to investigate the role of exosomal circular RNA (circRNAs) in regulating fibroblast growth factor 9 (FGF9) expression in DN through a competitive endogenous RNA (ceRNA) mechanism, and to reveal its potential therapeutic targets. Exosomes were isolated from serum of 3 healthy people and 3 patients with DN by ultra-fast centrifugation method, and the circRNA-miRNA-FGF9 regulatory network was constructed by combining high-throughput circRNA sequencing, bioinformatics analysis and weighted co-expression network (WGCNA). The results showed that the expression of circRNAs in serum exosomes of DN patients was significantly down-regulated, and hsa_circ_0006382 and hsa_circ_0019539 targeted the expression of FGF9 by binding to miR-34a-5p, miR-766-3p, miR-147a and miR-27a-3p. Further verification showed that the expression of FGF9 was decreased in renal tissues of DN patients (AUC=0.902), and its recombinant protein could inhibit the expression of α-SMA and vimentin in high glucose-induced NRK-52E cells, indicating that activation of the circRNA/miRNA-FGF9 network promotes the EMT of renal tubular epithelial cells. This study revealed for the first time the mechanism of the CircRNA-miRNA-FGF9 regulatory network in DN fibrosis, providing a theoretical basis for the development of diagnostic markers and targeted therapy strategies based on exosomal circRNA."

**2.** **The English language of this manuscript needs significant improvement before it is suitable for publication.**

Answer: Thanks for your suggestion, we have carried out the language polishing and obtained the language editing certificate. As follows:

**3.** **Line 144, the author should use adjusted p-value for multiple testing correction. The same issue is applicable to the p-value < 0.05 in Line 168. Please indicate which corrected method when performing multiple testing correction.**
Answer: Thank you very much for pointing out our mistake. We sincerely apologize for the inaccuracy in our description of the corrected P-value used in the circRNA differential expression analysis Method. In the figure, we used Padj.

The multiple hypothesis testing correction was performed to obtain the corrected P-value (Padj). The method used to calculate Padj was the Benjamini-Hochberg (BH) Method [1].

The set threshold of differential expression of circRNAs was | log_2_(Fold Change) | > 1 and Padj value < 0.05.

In the identification of differential expression analysis, the set threshold was |log_2_(Fold Change)| > 1 and Padj value < 0.05. The Padj was calculated using the Benjamini-Hochberg (BH) Method [1].

*[1] Benjamini, Y., & Hochberg, Y. (1995). Controlling the false discovery rate: apractical and powerful approach to multiple testing. Journal of the Royalstatistical society: series B (Methodological), 57(1), 289-300.*

We have revised the manuscript. As follows:

"According to the set threshold: | log_2_(Fold Change, FC) | > 1 and adjusted P- (Padj -) value < 0.05, circRNA differences between samples were screened. The corresponding Fold Change and Padj - value of up-regulated and down-regulated genes were obtained, and the volcano map was used for visualization. "

"The "limma" software package in R was used to identify differential expression genes (DEGs) between normal and DN samples, Padj < 0.05 and |log_2_FC| >1 gene was DEGs."

**4.** **In the ‘Results’ section, the results descriptions lack statistical data to supporting the findings. Especially for the first three paragraphs of the ‘Results’ section, I suggest the authors rewrite them. This is because the author actually only described 'what they have done in the study', but did not indicate 'what the results were'.**
Answer: Thank you for your suggestion. We have revised the first three paragraphs to include relevant statistical data according to your suggestion. And thank you for your careful suggestion again.

"Serum was obtained from 3 healthy individuals and 3 patients with DN, and exosomes were isolated and purified using the ultrafast centrifugation method. Transmission electron microscopy (TEM) was used to observe spherical particles with a diameter of 30-150 nm (Fig. 2A). The size of the particles analyzed by nanoparticle tracking analysis (NTA) technology was 81.35nm and the concentration was 1.93x109 particles/ml (Fig. 2B). The exosomes were extracted successfully. The purified exosomes were sequenced by circRNAs. The length distribution of circRNAs in each sample was statistically analyzed and plotted, the results showed that the length of most circRNAs was distributed between 149-15149nt (Fig. 2C). The type distribution map of the source regions of circRNAs showed circRNAs were derived from the splicing of exons, introns and intergenic regions (Fig. 2D). The chromosomal distribution map showed that the circRNA host genes were located on chromosome 1, 10, and 18. (Fig. 2E). The results of sample correlation heat map showed that the correlation between DN1 and DN2 was 0.997, indicating that the two samples were highly similar (Fig. 2F). The results of PCA diagram showed that the control group (red) and DN (blue) clustered on both sides of PC1 (46.97%), indicating that circRNA expression was significantly different between the two groups (Fig. 2G). The differential expression of circRNAs was analyzed, and the volcano map revealed that the expression of differentially expressed circRNAs was significantly decreased in DN (Fig. 2H). The clustering heatmap analysis revealed that circRNAs were generally green (low expression) in DN and red (high expression) in control group, indicating that low expression of circRNA might be related to DN (Fig. 2I). The top eight circRNAs were listed in descending order according to the | log_2_FC | value (Fig. 2J).

In order to further examine the biological function of differentially expressed circRNAs, GO, KEGG and Reactome were used to enrich the source genes of differentially expressed circleRNAs. Cellular component (CC) enrichment analysis revealed that these source genes were related to the N-terminal protein acetyltransferase complex, the site of DNA damage, the site of double-strand break, etc. Molecular function (MF) enrichment analysis revealed that these source genes were related to ATPase activator activity, RNA stem-loop binding, alpha-mannosidase activity, etc. (Fig. 3A). Similarly, KEGG pathway analysis revealed enrichment in protein processing in the endoplasmic reticulum (Padj < 0.01), porphyrin and chlorophyll metabolism (Padj < 0.05), N-Glycan biosynthesis (Padj < 0.05), Aminoacyl-tRNA biosynthesis (Padj < 0.05). (Fig. 3B). The results of Reactome enrichment analysis revealed that the circRNAs were related to tRNA modification in the nucleus and cytoplasm, and tRNA aminoacylation (Padj < 0.05), etc. (Fig. 3C).

The GSE30528 and GSE30529 dataset samples were combined after batch effect removal, including 19 DN tubule samples and 25 control samples. The data box plot after processing revealed that the median of each sample was almost on the same line, which meant the data were standardized successfully (Fig. 4A). PCA results showed PC1 (21%) and PC2 (16.9%), which indicated that samples were clustered according to DN and control group (Fig. 4B). The volcano map showed 202 upregulated genes and 64 downregulated genes in DN according to the analysis of DEGs (Fig. 4C). The heatmaps showed the top 10 upregulated (RARRES1, COL1A2, IGJ, IGLV1-44, LTF, C3, LYZ, ALOX5, CXCL6, MMP7) and downregulated genes (TYRP1, EGF, APOLD1, FOSB, NR4A3, ALB, ETNPPL, APOH, G6PC, CYP27B1) in DN (Fig. 4D)."

**5.** **Line 33, (DN) should be added to ‘diabetic nephropathy' in this sentence since DN abbreviation was used hereafter in the manuscript. On the contrary, Line 101, DN should be used rather than the full name for consistency purposes.**
Answer: Thank you for your reminding. According to your suggestion, we have added '(DN)' after 'diabetic nephropathy' in Line 33 and replaced the full name with 'DN' in Line 101 to ensure clarity and consistency throughout the manuscript.

**6.** **Lines 109-119, the author should provide the reference for the methods of exosome isolation. If no reference, the author should provide evidence that the exosome isolation process is successful in obtaining qualified exosomes for sequencing.**
Answer: Thank you for your suggestion. We have added references to the methods of exosome isolation in lines 109-119 of the manuscript.

*Théry C, Amigorena S, Raposo G, Clayton A. Isolation and characterization of exosomes from cell culture supernatants and biological fluids. Curr Protoc Cell Biol. 2006;Chapter 3:Unit 3.22. doi: 10.1002/0471143030.cb0322s30. PubMed PMID: 18228490.*

**7.** **Line 282, the statistical data in the parentheses seems to be wrong here based on Figure5D. The authors should rewrite this sentence to make it correct.**

Answer: Thank you for your reminding. We have corrected this information in the text to accurately reflect the data presented in Figure 5D. The revised sentence now correctly states the statistical findings.

"Further analysis of the correlation between gene membership (MM) and gene significance (GS) in the three modules revaled the positive correlation between MM and GS, in the pink module (MM and GS correlation = 0.34; *P* = 3E-07, Fig. 5D) "

**8.** **Lines 307-308, I suggest the authors move this description to 'Methods' section and make some adjustment appropriately. It would be confusing to organize the manuscript elements in wrong sections.**

Answer: Thank you very much for your careful review and valuable suggestions. We have moved the description from lines 307-308 to the 'Methods' section and made the necessary adjustments to ensure clarity and proper organization of the manuscript.

"The miRNA-specific binding sites of differentially expressed circRNAs were predicted using miRanda and psRobot. "

**9.** **The second paragraph of the ‘Discussion’ section should be moved to 'Introduction' section.**

Answer: Thank you for your valuable suggestion. We have moved the paragraph to the “Introduction” section and made appropriate adjustments to ensure it the smoothness of the content.

"Exosomes originate in the extracellular vesicles of endosome and are the most typical type of extracellular EVs. Exosomes can be isolated from a variety of mammalian cells and are present in almost all types of biological fluids, including blood, urine, prostate fluid and saliva. Exosomes contain a variety of signaling molecules, including proteins, DNA, mRNAs, miRNAs, long-chain non-coding RNAs, circRNAs, etc. They are the key mediators of paracrine function, and can fuse with the cell membrane, transfer nucleic acid substances and proteins to target cells, and mediate functions in cells.circRNAs are a class of non-coding RNAs (ncRNAs) produced by reverse splicing, which are highly stable in structure and evolutionarily conserved, and are stably enriched in exosomes. Exosomal circular RNAs (circRNAs) have the dual characteristics of exosome delivery function and circRNA transcriptional regulation, and have the potential to become DN biomarkers due to their high stability, expression specificity and easy detection. circRNAs can function as sponges for miRNAs, indirectly regulating mRNA expression, known as competitive endogenous RNA (ceRNA) function. The dysregulation of ceRNAs disrupts the balance of cellular processes and functions. circRNAs have become key regulators of a number of diseases, including cancer, cardiovascular disease, kidney disease etc.. Studies have shown that circRNAs have been detected in the blood of patients with DN. However, studies on the role of circulating exosomes-circRNAs in DN are limited. "

**Reviewer 2:** The manuscript investigates how circulating exosome-circRNAs mediate FGF9 downregulation via the ceRNA mechanism, exacerbating renal fibrosis in diabetic nephropathy (DN). The study uses high-throughput sequencing, bioinformatics analysis, and in vitro experiments to construct a circRNA-miRNA-FGF9 regulatory network, suggesting its role in fibrosis progression.

However, this paper is not good for publish because of the lack of sufficient validation and experimental depth.

**1.The study relies on only six human samples (3 DN, 3 controls), which is too small for meaningful conclusions.**
Answer: Thank you very much for your valuable comments. In the design of this study, we refer to an article in PNAS journal (1) for sample size requirements. It is the top journal in the field of nephrology and has a guiding role in our research. In this literature, the authors also used single-cell RNA sequencing for 2 sets of samples (3 diabetic nephropathy and 3 control samples), providing meaningful insights into early transcriptional changes in diabetic nephropathy. Therefore, two groups were included in our study, with three samples each for follow-up experiments.

The limited sample size might indeed have had an impact on the generality of the results. In the discussion section, we have discussed the limitations of a small sample size. In order to eliminate the uncertainty brought by it, we further verified it through in vitro cell experiment and animal experiment in this study. More samples would be collected in future studies to verify the generalizability of our results. We thank you again for your attention to this issue.

"In conclusion, in the present study, the circRNA-miRNA-FGF9 mRNA network was constructed by high-throughput sequencing of circulating exosome-circRNAs, miRNA binding site prediction and WGCNA co-expression analysis, and the role of the key gene, FGF9, in the EMT of renal tubular epithelial cells was validated in vitro. The findings presented herein may provide new evidence of the role of the ceRNA mechanisms of the circRNA-miRNA interaction in renal fibrosis in DN. However, the clinical sample in this study was limited. The limited sample size might indeed have had an impact on the generality of the results. In order to eliminate the uncertainty brought by it, we further verified it through in vitro cell experiment and animal experiment. More samples would be collected in future studies to verify the generalizability of our results. "

(1). *Wilson PC, Wu H, Kirita Y, Uchimura K, Ledru N, Rennke HG, et al.* *The single-cell transcriptomic landscape of early human diabetic nephropathy. Proc Natl Acad Sci U S A. 2019;116(39):19619-25. Epub 20190910. doi: 10.1073/pnas.1908706116. PubMed PMID: 31506348; PubMed Central PMCID: PMCPMC6765272.*

**2.** **Functional validation is weak, focusing only on α-SMA expression and cell viability.**

Answer: We sincerely appreciate the reviewer’s insightful comment. One of the core pathological features of diabetic nephropathy (DN) is renal tubular fibrosis, which is closely related to the epithelial-mesenchymal transition (EMT) of renal tubular epithelial cells, with α-SMA and vimentin being important markers of EMT (1). Therefore, in functional verification, we focus on the role of FGF9 in the fibrosis of renal tubular epithelial cells.

Preciously, in vitro experiments demonstrated that FGF9 significantly downregulated α-SMA expression in NRK-52E cells as previously confirmed by Western blot analysis, while CCK-8 assays validated its regulatory effects on cell viability. In this revision, we have additionally confirmed through Western blot that FGF9 reduces vimentin expression, and immunofluorescence staining further verified its inhibitory effect on α-SMA expression (Figure 1). The corresponding data have been incorporated into the revised Figure 8 of this submission. The relevant methods, results and figure legends have all been incorporated into the manuscript.

**Figure 1:**

**Figure 1** (A) Representative western blots showed that vimentin protein expression after FGF9 treatment. (B) Summarized data of western blots showed that vimentin protein expression was decreased after FGF9 treatment. (C) Representative images of immunofluorescence staining for a-SMA (green) and DAPI (blue) (Scale bars: 75 μm). * *P* < 0.05, ** *P* < 0.01, *** *P* < 0.001.

"**Method**

**Immunofluorescence of α-SMA**

NRK-52E cells were seeded with a density of 10^6^cells/ well and incubated at 37°C, 5% CO_2_ for 24h. The cells were fixed with 4% paraformaldehyde, permeabilized with 0.1% Triton X-100, and blocked with nonspecific antigens with 3% BSA. The cells were incubated with α-SMA antibody (1:200, product number: #19245T, Cell Signaling Technology, Inc.) overnight at 4°C, followed by Anti-rabbit lgG Fab 2 Alexa Fluor 488 (Cat. #4412, 1:200, Cell Signaling Technology, Inc.) at room tem-perature for a further 2h, and the fluorescent nuclear DAPI stain (Beyotime, Beijing, China) for 5min. Then fluorescent images were observed under a confocal laser scanning microscope (ZEISS, Germany).

**Western blot**

Primary antibodies against vimentin (1:4000, product number: 10366-1-AP, ProteinTech Group, Inc.)

**Results**

In NRK-52E cells, the expression of α-SMA and vimentin was significantly decreased after FGF9 treatment. (Fig. 8G, J). Immunofluorescence staining revealed that a-SMA expression level was obviously increased in HG-treated cells compared with normal glucose, a-SMA expression decreased in NRK-52 cells of HG + FGF9 group (Fig. 8L), indicating that FGF9 could alleviate EMT in NRK-52E cells. * *p* < 0.05, *** *p* < 0.001."

In in vivo experiments, another study in our group is currently conducting animal experiments on the therapeutic effects of FGF9 on animal models of diabetes. We used db/db Mouse Model of Type 2 Diabetes. db/db mice develop diabetes spontaneously. 20 weeks db/db mice were given FGF9 (40ug/kg) intravenously once every two days for a total of four injections. Blood glucose was measured by taking blood from the tail vein. It showed that FGF9 could reduce the blood glucose in db/db mice (Figure 2). However, because the animal experiments have not been completed, this was only a preliminary conclusion. So we did not include this result in the manuscript.

**Figure 2:**

**Figure 2** Blood glucose of db/db mice was significantly higher than that of db/m mice. Compared with db/db mice, the blood glucose reduced in db/db mice with FGF9 treatment.

(1). *Yang YL, Xue M, Jia YJ, Hu F, Zheng ZJ, Wang L, et al. Long noncoding RNA NEAT1 is involved in the protective effect of Klotho on renal tubular epithelial cells in diabetic kidney disease through the ERK1/2 signaling pathway. Exp Mol Med. 2020;52(2):266-80. Epub 20200214. doi: 10.1038/s12276-020-0381-5. PubMed PMID: 32054986; PubMed Central PMCID: PMCPMC7062691.*

**3. miRNA binding predictions lack experimental validation (e.g., overexpression/inhibition studies)**

Answer: We appreciate the reviewer’s insightful comment regarding the need for experimental validation of miRNA binding predictions. In this study, While we constructed a circRNA-miRNA-FGF9 regulatory network based on bioinformatics predictions, the current work emphasizes the functional validation of FGF9 itself, demonstrating its anti-fibrotic effects through the downregulation of α-SMA and vimentin.

We fully agree that further experimental validation of miRNA interactions (e.g., miRNA overexpression/inhibition studies) would strengthen the mechanistic insights. Such investigations are indeed planned as part of our future research. We believe the present findings on FGF9’s role in EMT inhibition provide a solid foundation for these follow-up studies.Thank you for your valuable suggestion.
